# Reaction Optimization for Greener Chemistry with a Comprehensive Spreadsheet Tool

**DOI:** 10.3390/molecules27238427

**Published:** 2022-12-02

**Authors:** Daniel M. Day, Thomas J. Farmer, Joe Granelli, Janice H. Lofthouse, Julie Lynch, Con R. McElroy, James Sherwood, Seishi Shimizu, James H. Clark

**Affiliations:** 1 Green Chemistry Centre of Excellence, Department of Chemistry, University of York, Heslington YO10 5DD, UK; 2 York Structural Biology Laboratory, Department of Chemistry, University of York, Heslington YO10 5DD, UK

**Keywords:** green chemistry, green metrics, kinetics, Michael addition, solvents, Variable Time Normalization Analysis

## Abstract

Green chemistry places an emphasis on safer chemicals, waste reduction, and efficiency. Processes should be optimized with green chemistry at the forefront of decision making, embedded into research at the earliest stage. To assist in this endeavor, we present a spreadsheet that can be used to interpret reaction kinetics via Variable Time Normalization Analysis (VTNA), understand solvent effects with linear solvation energy relationships (LSER), and calculate solvent greenness. With this information, new reaction conditions can be explored in silico, calculating product conversions and green chemistry metrics prior to experiments. The application of this tool was validated with literature case studies. Reaction performance was predicted and then confirmed experimentally for examples of aza-Michael addition, Michael addition, and an amidation. The combined analytical package presented herein permits a thorough examination of chemical reactions, so that the variables that control reaction chemistry can be understood, optimized, and made greener for research and education purposes.

## 1. Introduction

The twelve principles of Green Chemistry promote the reduction of hazards, energy use and waste from chemical processes [1]. The optimization of reaction conditions and reagents to achieve the objectives of green chemistry requires a fundamental understanding of chemical reactions [2,3]. Processes should be monitored during their development and data extracted to inform reaction optimization. This makes data management and interpretation using spreadsheets a valuable asset and providing adequate training to scientists in this regard is crucial [4]. The greenness of a reaction is often evaluated with metrics. To do so, customized spreadsheets are available [5,6,7]. The yield of a reaction has a strong influence on mass-based green chemistry metrics. The rate of reaction also impacts energy use; a relatively fast reaction can be performed for a shorter time period or at a lower temperature and achieve the same yield as a relatively slow reaction. Therefore, reaction kinetics are extremely valuable to evaluate the efficiency of a reaction. Variable Time Normalization Analysis (VTNA) has proven to be a valuable technique to determine reaction orders without requiring a thorough understanding of the mathematical derivations of potentially complex rate orders [8,9,10]. A spreadsheet is generally used to perform VTNA, making the mass utilization of a process easy to monitor and interpret.

The rate of a reaction can vary dramatically with the polarity of the solvent [11]. It is therefore advantageous to understand the solvent properties that enhance the performance of the reaction. At the same time, solvent use presents a high safety risk because many solvents are volatile and flammable. Solvents are usually responsible for the majority of the mass in a reaction, increasing the potential for exposure and exacerbating the risk posed by any hazards. Therefore, green yet high-performance solvents are desirable. Tools for solvent selection include general-purpose tables ranking entries in order of greenness [12], and reaction-specific summaries of the yields obtained in different solvents under certain conditions [13,14]. To fully understand solvent effects, elements of both these tools are required. Quantitative multiple linear regression analysis is used to correlate solvent polarity with chemical phenomena, usually rate constants [15]. The resulting linear solvation energy relationship (LSER) can be used to understand the mechanism of the reaction. Since the advent of green chemistry, analysis by LSER has gained a new use as a means of identifying the properties of high-performance solvents in an application [16]. As with green metrics and kinetic analysis, a spreadsheet or other data processing software is required to create and interpret a LSER, and the raw data that is required is the same, namely the reaction component concentrations at defined time intervals. Combining these related concepts into a single tool would provide a thorough understanding of reactions so that they might be optimized to be greener.

**Scheme 1:**
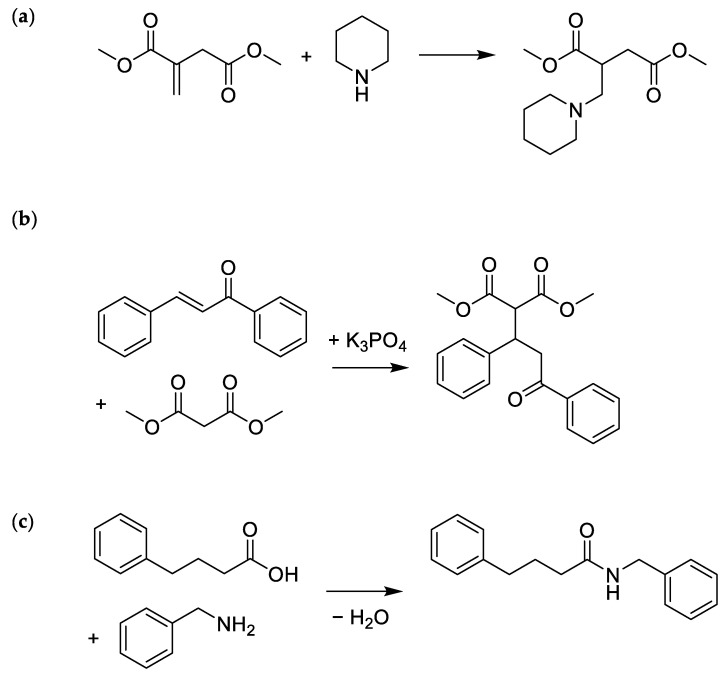
Reaction case studies. (**a**) Aza-Michael addition between dimethyl itaconate and piperidine; (**b**) Michael addition between *trans*-chalcone and dimethyl malonate; (**c**) Amidation between 4-phenylbutyric acid and benzylamine.

In this work, a spreadsheet has been designed to assist the combined processing of kinetic data by VTNA, generate LSERs, and shortlist suitable green solvents and reaction conditions. The Excel spreadsheet can be used to assist chemistry students with their physical or physical organic chemistry laboratory classes but is also of general use to any chemist optimizing a reaction. The functions of the spreadsheet are demonstrated in this article with examples of the Michael addition [16], aza-Michael addition [17], and an amidation (see Scheme 1) [18].

## 2. Results

The reaction optimization spreadsheet is divided into worksheets with different functions. Two versions are available as Supplementary Materials S1 and S2. Reaction data (in the form of reaction component concentrations at specified times) must be provided. Data only documenting (product) conversion as a function of time can also be used, in which case the concentrations of reactants and catalysts are estimated from the initial conditions. Then, the data can be processed according to the functions summarized in Table 1. Important functions are described subsequently in more detail with the use of case studies. Additional datasets were analyzed to ensure the robustness of the spreadsheet. These were the aza-Michael addition of dimethyl itaconate and dibutylamine [17], the isomerization of dimethyl itaconate in the presence of triethylamine [17], and a Fischer esterification to produce benzyl acetate [19]. All the data sets are provided in Supplementary Material S3.

### 2.1. Reaction Optimisation Spreadsheet Function Tutorial

The reaction between dimethyl itaconate and amines (i.e., piperidine and dibutylamine, see Scheme 1a) was previously studied extensively, investigating the order of reaction, rate constants at different temperatures and in different solvents, as well as solvent effects, activation parameters (ΔH^‡^ and ΔS^‡^), and the competitive base catalyzed isomerization of dimethyl itaconate to dimethyl mesaconate [17,20]. This information can all be inferred from the measurement of reactant and product concentrations at timed intervals (in this case using ^1^H NMR spectroscopy) but each facet of the investigation requires its own data processing and interpretation. It was realized that because the raw data is the same, a single tool could derive all the desired information.

**Figure 1:**
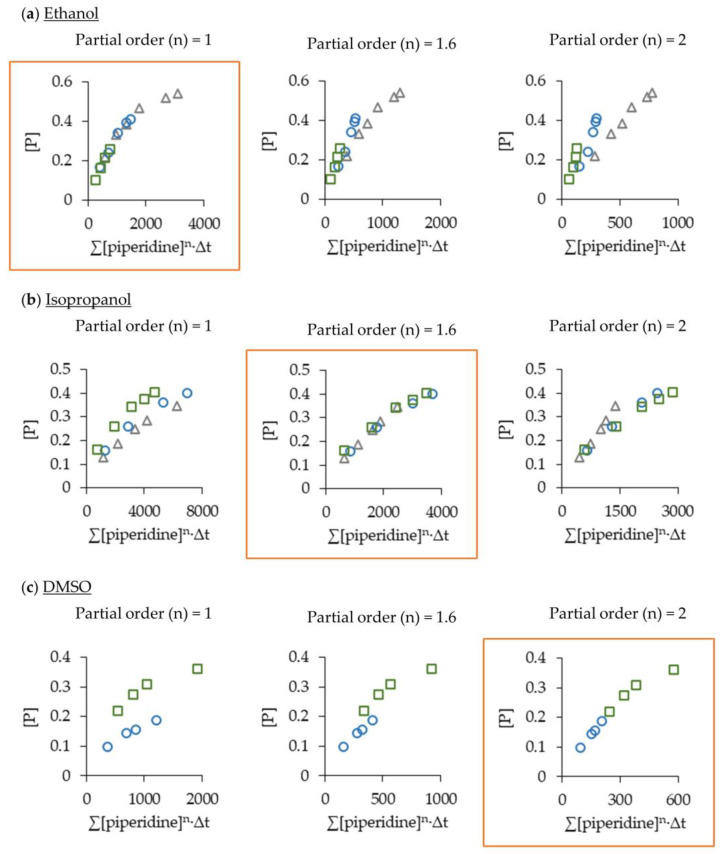
Variable Time Normalization Analysis of an aza-Michael addition to establish the partial order of reaction with respect to piperidine concentration. (**a**) In ethanol; (**b**) In isopropanol; (**c**) In dimethyl sulfoxide (DMSO). Data symbols refer to separate experiments performed at different initial concentrations (see Supplementary Material S3). Graphs in a box correspond to the correct partial reaction order (n = 1, 1.6, or 2).

The aza-Michael addition between dimethyl itaconate and piperidine experiences different orders of reaction depending on the solvent. With VTNA, the order of reaction was always found to be 1 with respect to dimethyl itaconate. The mechanism can be bimolecular in protic solvents but is generally trimolecular (second order in amine). The trimolecular reaction occurs when a second amine molecule assists with proton transfer during the rate-limiting addition step [17]. In protic solvents, the solvent can perform this role. Hence, due to the large molar excess of solvent, pseudo-second order kinetics are observed. In isopropanol, a non-integer order of reaction is observed (1.6 with respect to piperidine). In this unique circumstance, the observed rate of reaction is similar for both the solvent- and amine-assisted mechanisms. The data fitting exercise to obtain the orders of reaction can be completed with the reaction optimization spreadsheet. The reaction conversion as a function of changing piperidine concentration ([piperidine]) and time (t) is shown in Figure 1. The data from reactions with different initial reactant concentrations overlap when the correct order of the reaction is inputted [8]. The reaction optimization spreadsheet guides the user to test different potential reaction orders (Supplementary Materials S1 and S2) and automatically calculates the resultant rate constant.

**Figure 2:**
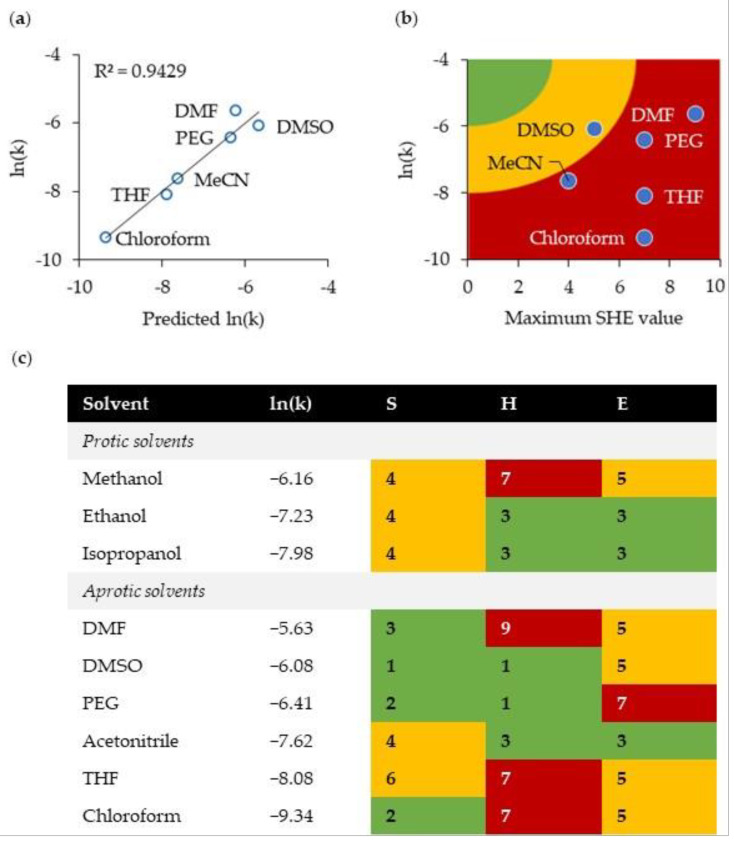
Solvent selection of an aza-Michael addition. (**a**) Linear solvation energy relationship for the trimolecular mechanism; (**b**) Comparison between the rate constant and solvent greenness (color scheme is for guidance); (**c**) Summary of solvent greenness scores (protic solvents do not facilitate the trimolecular reaction, PEG is a weak hydrogen bond donor). DMF, *N*,*N*-dimethyl formamide; DMSO, dimethyl sulfoxide; PEG, polyethylene glycol-400; THF, tetrahydrofuran.

Once the order of reaction and rate constants are determined, the solvent effect is deduced from a LSER. The relationship between ln(k) and solvent polarity is only valid for the set of the solvents supporting the same order of reaction at the same temperature. The output of the reaction optimization spreadsheet is given in Figure 2 for the trimolecular aza-Michael reaction at 30 °C. Kamlet-Abboud-Taft solvatochromic parameters were used to describe solvent polarity: α is hydrogen bond donating ability, β is hydrogen bond accepting ability, π* is dipolarity/polarizability [21]. Molar volume (V_m_) is also included as a potential variable in the LSER to account for cavitation effects. The spreadsheet user can determine the coefficients to produce a statistically relevant correlation. For the trimolecular reaction of dimethyl itaconate and piperidine, the reaction is accelerated by polar, hydrogen bond accepting solvents (Equation (1)) [17]. It is probable that the positive correlation with β reflects stabilization of the proton transfer, while polar or polarizable solvents stabilize the charge delocalization of the activated complex. The rate constants predicted using Equation 1 are plotted against experimental data in Figure 2a.

ln(k) = −12.1 + 3.1β + 4.2π*(1)

To decide upon the greenest conditions, an efficient solvent (i.e., one producing high conversions in a short timescale) with few hazards is beneficial. The reaction optimization spreadsheet assists solvent selection by plotting a chart of ln(k) against solvent greenness. The latter is determined with the CHEM21 solvent selection guide [22]. Safety (S), health (H), and environment (E) scales from 1 (greenest) to 10 (most hazardous) are available. To obtain a single scale (for ease of interpretation), either the sum of the three scales is used, or the worst (highest) score is taken to compare to ln(k) (Figure 2b). Dimethyl sulfoxide (DMSO) is arguably the optimum solvent, with a rate constant second only to the reprotoxic *N*,*N*-dimethyl formamide (DMF). Solvent greenness and rate constants are compared in Figure 2c. Clearly, there are solvents with a superior environmental health and safety profile to DMSO, which is designated as a ‘problematic’ solvent [22]. There are concerns over the ability of DMSO to pass through the skin barrier and transport substances into the body [23], as well as the propensity of DMSO to decompose at elevated temperatures [24]. Alternatives to DMSO for aza-Michael reactions are discussed in Section 2.2.

### 2.2. Optimising the Aza-Michael Reaction

Green chemistry metrics are an important consideration when determining the most beneficial reaction conditions. The reaction optimizer spreadsheet (Supplementary Materials S1 and S2) reports conversion and atom economy, and on that basis calculates reaction mass efficiency (RME) and optimum efficiency [6], optimum efficiency being the quotient of RME when divided by atom economy.

**Figure 3:**
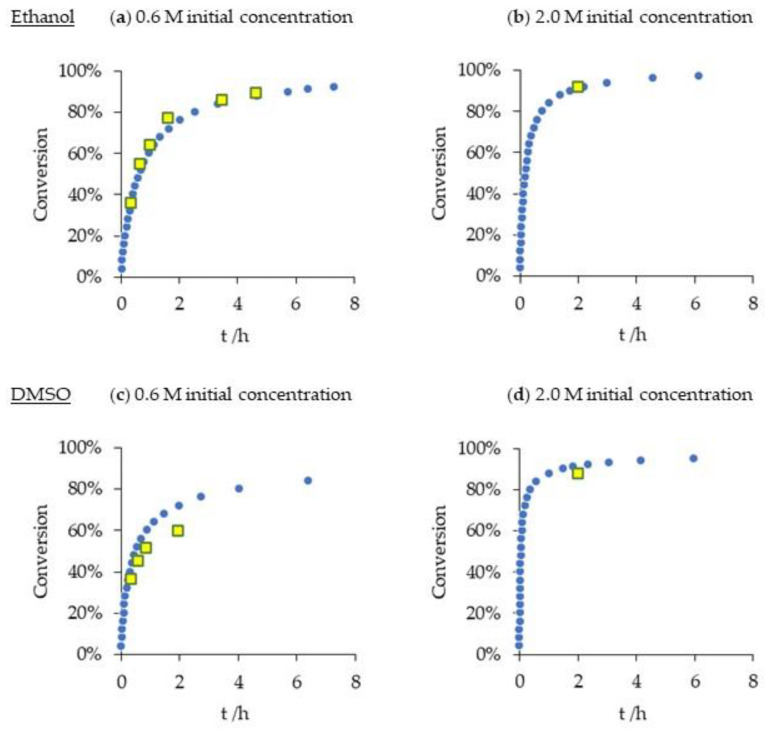
Aza-Michael reaction optimization. Blue (circular) data points are predicted conversions. Yellow (square) data points represent experimental data. (**a**) Reaction in ethanol with initial reactant concentrations of 0.6 M; (**b**) Reaction in ethanol with initial reactant concentrations of 2.0 M; (**c**) Reaction in dimethyl sulfoxide (DMSO) with initial reactant concentrations of 0.6 M; (**d**) Reaction in DMSO with initial reactant concentrations of 2.0 M.

It was shown that DMSO is an appropriate solvent for the reaction between dimethyl itaconate and piperidine as it produces a fast rate of reaction (via the trimolecular reaction mechanism). A conversion of 60% was obtained after 2 h at 30 °C from initial reactant concentrations of 0.6 M in DMSO. The atom economy of Michael-type addition reactions is 100%. RME and optimum efficiency are equivalent to the yield when equimolar reactant loadings are used. However, protic solvents that promote the bimolecular reaction should also be considered. The rate constant is smaller in magnitude in ethanol (k = 7.25 × 10^−4^ dm^3^·mol^−1^·s^−1^) than it is in DMSO (k = 2.29 × 10^−3^ dm^6^·mol^−2^·s^−1^) at 30 °C. However, the observed rate of reaction is dependent on the concentration of piperidine and its different partial orders of reaction in the two solvents (Figure 1). This means the reaction conditions can be tuned to favor ethanol, which is recommended in the CHEM21 solvent selection guide [22]. To further enhance the conversion and the associated mass-based green chemistry metrics, the initial concentration of reactants can be increased, or the reaction temperature, or both. The reaction optimizer spreadsheet can be used to predict conversion as a function of time in any solvent that meets the requirement of the LSER (same mechanism, same temperature). If the initial concentration of both reactants is 2 M, a conversion of 90% is anticipated in DMSO and ethanol after 2 h at 30 °C (Figure 3). This was verified experimentally (Figure 3b,d). The actual conversion in DMSO is slightly short of the prediction at 88% due to isomerization of dimethyl itaconate to dimethyl mesaconate [17,20], lowering the availability of the intended reactant. The suppressed conversions in DMSO are evident in Figure 3c. Isomerization is suppressed in ethanol, and a conversion of 92% was observed after 2 h (Figure 3b). Calculated RME and optimum efficiency are also 92%, improved from 60% as observed in the initial reaction conditions (Figure 3c).

### 2.3. Green Solvent for the Michael Reaction

**Figure 4:**
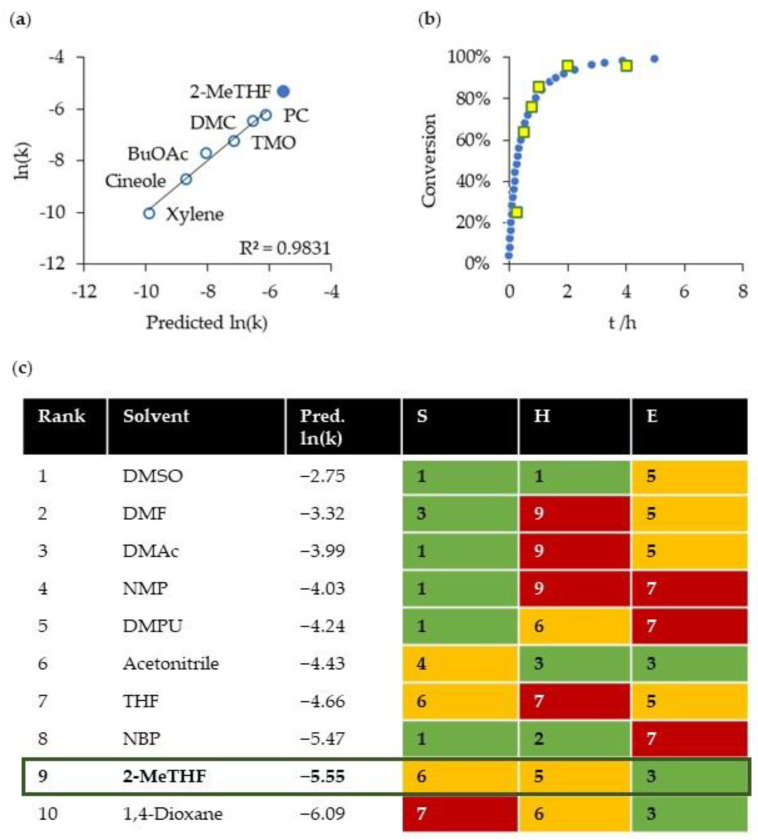
Solvent optimization for a Michael addition. (**a**) Linear solvation energy relationship calculated prior to experiment in 2-methyltetrahydrofuran (2-MeTHF), with data for 2-MeTHF added (solid fill data point); (**b**) Blue (circular) data points representing predicted conversions and yellow (square) data points represent experimental data for the optimized reaction conditions; (**c**) Solvent selection table obtained from the reaction optimization spreadsheet. Bio-based 2-MeTHF is emphasized in the box. Other abbreviations: DMSO, dimethyl sulfoxide; DMF, *N*,*N*-dimethyl formamide; DMAc, *N*,*N*-dimethyl acetamide; NMP, *N*-methyl pyrrolidone; DMPU, dimethyl propylene urea; THF, tetrahydrofuran; NBP, *N*-butyl pyrrolidone.

To further test the reaction optimization spreadsheet, kinetic data for a conventional Michael addition between dimethyl malonate and trans-chalcone was obtained from the literature (Scheme 1b) [16]. The reactions were performed at the ambient temperature in *p*-xylene, cineole (eucalyptol), *n*-butyl acetate (BuOAc), 2,2,5,5-tetramethyloxolane (TMO), dimethyl carbonate (DMC), and propylene carbonate (PC). The LSER suggests the reaction is accelerated by hydrogen bond accepting solvents with a small molar volume (Equation (2) and Figure 4a).

ln(k) = −4.5 + 7.4β − 0.0052V_m_ (2)

The reaction optimization spreadsheet (Supplementary Materials S1 and S2) can be used to provide a list of suitable solvents (Figure 4c). The ideal physical properties are possessed by dipolar aprotic solvents, including DMSO, DMF, and *N*,*N*-dimethyl acetamide (DMAc). The nitrogen-containing solvents have chronic toxicity issues [22], but further down the list is 2-methyltetrahydrofuran (2-MeTHF), a bio-based solvent with low environmental hazards [22,25]. No other solvents in the shortlist are bio-based. The predicted rate constant of the reaction in 2-MeTHF was ln(k) = −5.55, while the experimental value was found to be −5.30 (Figure 4b). This is a larger rate constant than produced by any other of the experimentally verified solvents (Figure 4a).

### 2.4. Amidation

A final data set was scrutinized using the reaction optimizer spreadsheet. Kinetic data for the thermally activated reaction between 4-phenylbutyric acid and benzylamine to form the corresponding amide *N*-benzyl (4-phenyl)butanamide in toluene at 100 °C was reanalyzed (Scheme 1c) [18]. It was revealed that the rate equation of the previously assumed bimolecular reaction actually has a less than first order relationship with respect to the concentration of benzylamine. Using VNTA it appears that the reaction order with respect to benzylamine is approximately 0.25. Previous studies of amidation in the absence of catalysts or coupling agents at elevated temperatures have implied an acid anhydride is formed prior to acylation of the amine [26]. If rate-limiting, the concentration of amine would be irrelevant to the rate of reaction, and instead second order with respect to carboxylic acid concentration. This is not consistent with what was observed experimentally in the present example. In the presence of boron-based catalysts, amidations experience a negative order of reaction with respect to the carboxylic acid [27]. This is the result of competing reactions, such as the deactivation of catalyst, or amine by salt formation. Ultimately it is unclear what the exact mechanism is, but it would appear to be complicated by the pre-equilibrium established by the acid-base interaction between the two reactants.

**Figure 5:**
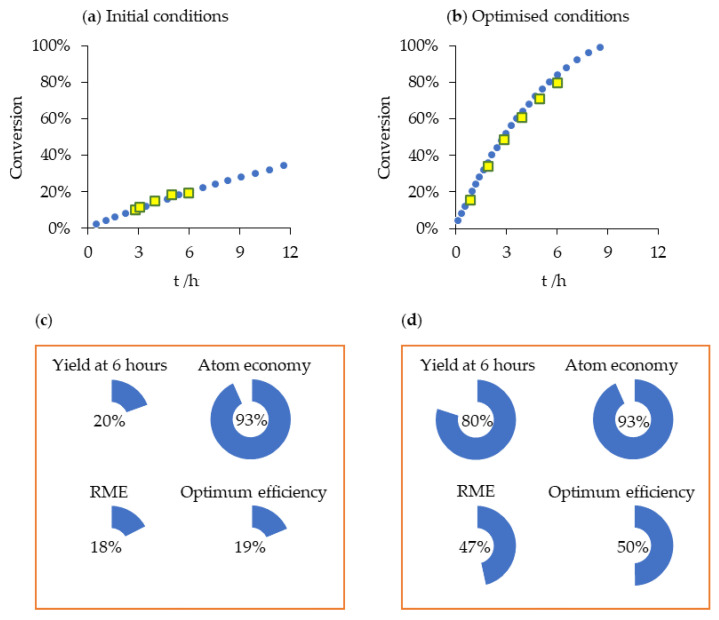
Rate acceleration of an amidation reaction in toluene. (**a**) Initial conditions (0.5 M 4-phenylbutyric acid, 0.55 M benzylamine, 100 °C); (**b**) Optimized conditions (1.1 M 4-phenylbutyric acid, 0.55 M benzylamine, 111 °C). Blue (circular) data points are predicted conversions. Yellow (square) data points represent experimental data. Metrics (**c**) prior to; and (**d**) after optimization are displayed.

Using the revised rate equation, first order with respect to 4-phenylbutyric acid, 0.25th order with respect to benzylamine, rate constants and activation parameters were recalculated. These are automatic features of the reaction optimizer spreadsheet (Supplementary Materials S1 and S2), obtained directly from the VTNA. It was then possible to suggest improved reaction conditions. There is little benefit in using an excess of benzylamine, while the concentration of 4-phenylbutyric acid is proportional to the rate of reaction. Therefore, an excess of 4-phenylbutyric acid was applied (2 equivalents) and the temperature increased to reflux in toluene. The rate constant was predicted via the Erying equation, and in turn used to calculate the theoretical conversions at set time intervals. Experimental data was obtained that matched the predicted conversions (Figure 5b). The mass-based metrics are improved by the excess of 4-phenylbutyric acid, despite the inevitable waste it creates (Figure 5d). This is because of the vastly improved yield, from 20% to 80%.

## 3. Discussion

The combined kinetic analysis, solvent selection, and metric evaluation functions of the reaction optimizer spreadsheet enable a fuller appreciation of the greenness and efficiency of a reaction than when only one of these techniques is used. Through quantitative solvent effect correlations and the use of the Eyring equation to deduce activation parameters, this analysis can be used to plan reaction conditions before they are attempted in the laboratory. This was tested with three reactions and the predicted reaction rates and conversions were achieved experimentally. In the case of the aza-Michael reaction in DMSO, experimental conversions were lower than predicted due to a competing side-reaction. The ability to describe concurrent reactions in the reaction optimizer spreadsheet was considered but ultimately not pursued because of the extra complexity. To accurately model a side-reaction, separate studies may be required, increasing the experimental burden. In the remaining case studies, the prediction of product conversion with time was reliable. The ability to visualize the (predicted) rate of reaction in unfamiliar conditions helps to quickly differentiate between possible optimization strategies.

The theory and format of all the tools in the reaction optimizer spreadsheet are well established, so it must be asked whether it is helpful to offer the analysis package at all. The reaction optimizer spreadsheet has been shown to be a convenient tool to predict reaction efficiency and waste, but researchers can gain helpful knowledge and transferable skills by designing and managing their own data analysis systems. Rubin and Abrams [4] advocate for students to perform quantitative analysis with a spreadsheet of their own design in order to appreciate the underlying theory. Conversely, the purpose of applied science such as green chemistry is not to teach fundamental principles but to equip scientists with the tools to affect change. The reaction optimization spreadsheet is suitable for users not familiar with the mathematical basis of VTNA, LSER, or mass-based green chemistry metrics, making these useful functions accessible to a more diverse array of practitioners. For experts in the aforementioned techniques, the user must be aware of the limitations of the reaction optimizer spreadsheet. The functions are simplified to provide the most common types of solvent and kinetic analyses but will not be suitable for every reaction (for example, if more than three reactants and catalysts are required, or the reactions are in the gas phase or solvent-free). The LSER function can only describe ln(k) but it is possible to correlate solvent properties with equilibria, selectivity, and other chemical phenomena as well [11]. Some aspects of green chemistry are not (directly) assessed with the reaction optimizer spreadsheet, such as energy use, feedstock origin, or the toxicity of reactants. A balance was sought so that the reaction optimization spreadsheet offers the most common and helpful data analysis for green reaction chemistry, without containing an overwhelming number of complex functions.

## 4. Conclusions

This tool succinctly combines several well-established methods to compare and optimize reactions, enabling a holistic approach for practitioners who are unable or who do not desire to design their own bespoke data management system. Rate constants and conversion plots can be calculated alongside green chemistry metrics. The accuracy of the predictive elements of the spreadsheet were shown to be high, permitting the optimization of Michael additions and amidations.

## 5. Materials and Methods

The reaction optimization spreadsheet has been made available as a Microsoft 365 compatible file (Supplementary Material S1) and a backwards compatible file with certain formula replaced but with less functionality (Supplementary Material S2). The datasets used to demonstrate the spreadsheet have also been provided in a separate file (Supplementary Material S3). All experimental procedures have previously been reported, and new data was obtained using those same literature methods [16,17,18]. Details are presented in Supplementary Material S4.

## Figures and Tables

**Table 1:** Summary of reaction optimization spreadsheet functions.

Worksheet	Function
Data entry	Kinetic data measurements are added. In the Microsoft 365 compatible spreadsheet (Supplementary Material S1), repeat experiments are automatically combined.
Conversions	Product conversion as a function of time is displayed for selected reactions (maximum of five).
Kinetics	Orders of reaction are determined using VTNA. Rate constants (k) are calculated for each experiment.
Activation parameters	The enthalpy and entropy of activation are calculated for data sets with variable temperatures.
Solvent effects	The solvent effect describing the rate of reaction is calculated as a LSER.
Solvent selection	High performing solvents are predicted based on the calculated rate constant.
Solvent list	Tabulated solvent properties. The greenness of solvents can be calculated on this worksheet also.
Metrics	The product conversion at a set time is predicted based on the solvent and temperature and initial concentration of reactants. The green metrics describing the reaction are shown (atom economy, reaction mass efficiency, and optimum efficiency).

## Data Availability

All data is freely provided as Supplementary Materials.

## Supplementary Materials

The following supporting information can be downloaded at: https://www.mdpi.com/article/10.3390/molecules27238427/s1, Supplementary Material S1: Microsoft 365 compatible reaction optimization spreadsheet; Supplementary Material S2: reaction optimization spreadsheet (for Microsoft Excel 2016 and Excel 2019); Supplementary Material S3: Reaction case study data (component concentrations at given times); Supplementary Material S4: a guide to use the spreadsheets and additional experimental information. Additionally, the reaction optimizer spreadsheets will be maintained and remain openly available in Zenodo at https://doi.org/10.5281/zenodo.7267827 (accessed on 31 October 2022).
