# Supplementary material for: Reaction Optimization for Greener Chemistry with a Comprehensive Spreadsheet Tool
Source: Molecules. 2022 Dec 2;27(23):8427. doi: 10.3390/molecules27238427 (PMC9738638; doi:10.3390/molecules27238427)
Supplement: Supplementary file 1 [file molecules-27-08427-s001.zip › S4 Tutorial and additional information.pdf]

Supplementary Material:

Reaction optimization for greener chemistry with a comprehensive spreadsheet tool

Daniel M. Day, Thomas J. Farmer, Joe Granelli, Janice H. Lofthouse, Julie Lynch, Con R. McElroy, James Sherwood, Seishi Shimizu, and James H. Clark

Preface

The most up-to-date version of the reaction optimizer spreadsheet is publically available at https://doi.org/10.5281/zenodo.7267827. This user guide contains annotated screenshots from version 1 of the Microsoft 365 compatible reaction optimizer spreadsheet (Supplementary material S1).

1. Operating the reaction optimisation spreadsheet

The main purposes of the reaction optimisation spreadsheet are to determine kinetic data from experiment (orders of reaction and rate constants, activation parameters if applicable), solvent effects, and thus predict high performance solvents. Then the greenness of reactions can be measured with mass-based metrics.

There are 2 versions of the Excel spreadsheet. The Microsoft 365 compatible version is recommended (Supplementary material S1). Data that is not in chronological order and repeat experiments are tolerated. If you are using an older version of Excel (prior to Microsoft 365) some functions will not work in this file, and you are recommended to use the backwards compatible version of the reaction optimisation spreadsheet (Supplementary material S2).

When using the spreadsheet, editable cells are coloured yellow. Annotations are present for further assistance. A more comprehensive guide is provided below (Section 1.2).

1.1 Data sets

The data that was used to test the reaction optimiser spreadsheet is listed below. These data sets have been provided in a separate spreadsheet.

1. The aza-Michael addition of dimethyl itaconate and piperidine [1].
2. The aza-Michael addition of dimethyl itaconate and dibutylamine [1].
3. Isomerisation of dimethyl itaconate to dimethyl mesaconate [1]
4. The Fischer esterification of benzyl alcohol and acetic acid catalysed by *p*-cymenesulphonic acid [2].
5. Amidation between 4-phenylbutyric acid and benzylamine [3].
6. The Michael addition between *trans*-chalcone and dimethyl malonate [4].

1.2 User guide

The following guide is divided into sections corresponding to the worksheets in the spreadsheet.

Data entry: blank sheet

Figure S1a. Add the names of the reagents (reactants and catalysts, maximum of three).
Figure S1b. Add the name of the product.
Figure S1c. If a catalyst is included, change the adjacent cell to Y (yes).
Figure S1d. Add data points. Time and product concentration are required. Add the initial conditions (t = 0 data point) to start each experiment, which must include the initial concentrations of reagents. When t > 0, reagent concentrations are not required (they will be estimated from the product concentration if unavailable). Repeat dataset can be added to the Microsoft 365 version. To use the backward compatible spreadsheet, please average data prior to submission and ensure timed data points are listed chronologically for each experiment.
Figure S1e. Each t = 0 data point will require a temperature to represent the data set. You will be prompted to add this with a yellow cell.
Figure S1f. Each t = 0 data point will require a solvent to represent the data set. You will be prompted to add this by the appearance of a yellow cell.

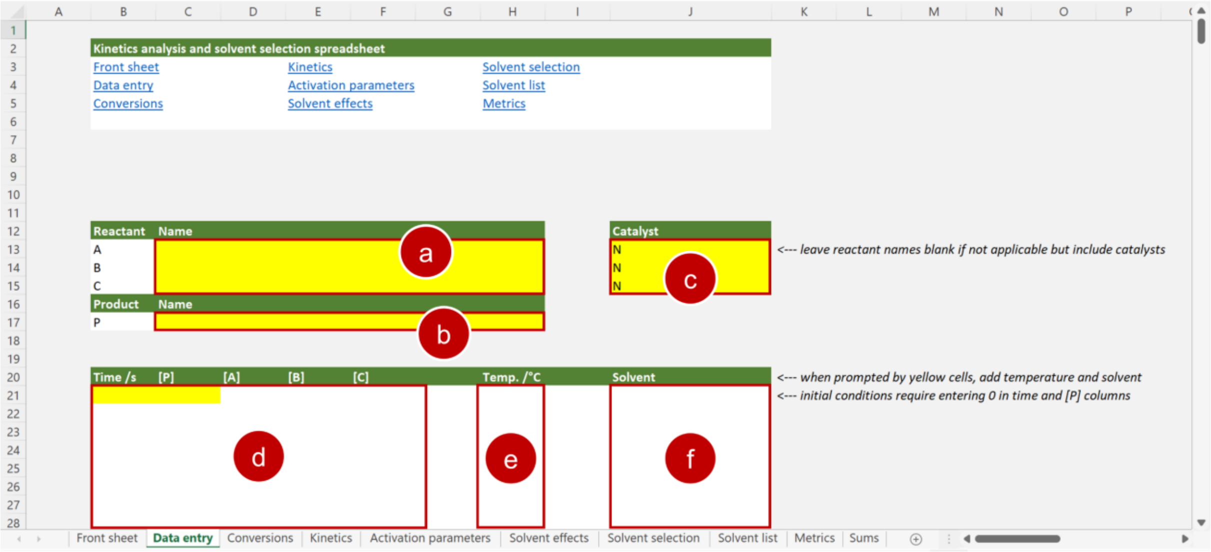

Figure S1. A screenshot of the Data entry worksheet, prior to data entry.

Data entry: completed sheet

The data set for the reaction between dimethyl itaconate and piperidine is shown in Figure S2. This data set will be used subsequently to illustrate the functions of the spreadsheet.

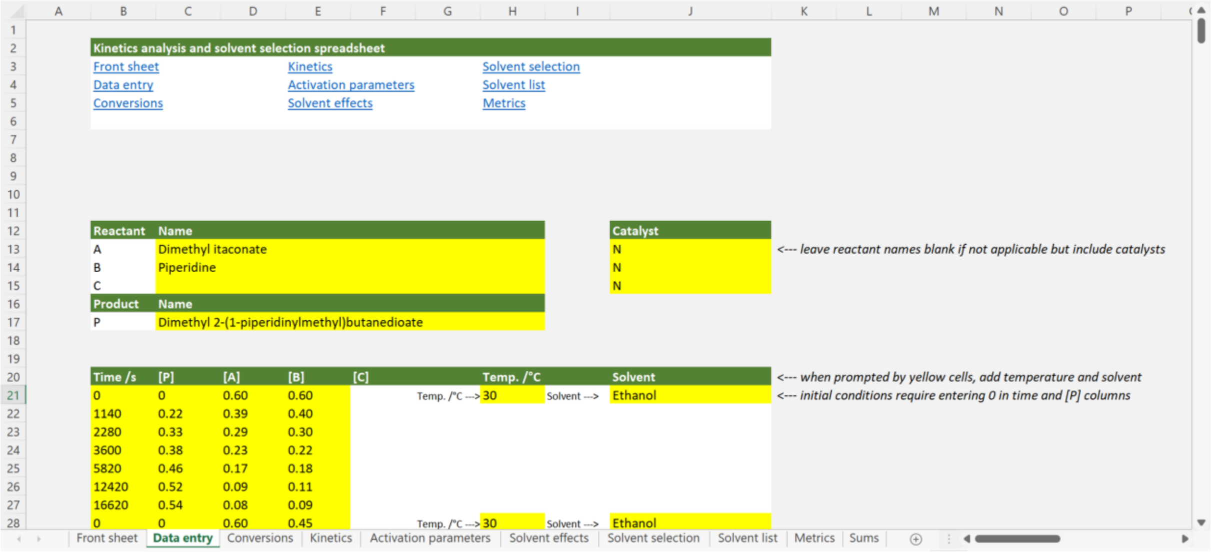

Kinetics analysis and solvent selection spreadsheet

Front sheet	Kinetics	Solvent selection
Data entry	Activation parameters	Solvent list
Conversions	Solvent effects	Metrics

Reactant	Name	Catalyst
A	Dimethyl itaconate	N
B	Piperidine	N
C		N
**Product**	**Name**	
P	Dimethyl 2-(1-piperidinylmethyl)butanedioate	

<--- leave reactant names blank if not applicable but include catalysts

Time /s	[P]	[A]	[B]	[C]		Temp. /°C		Solvent
0	0	0.60	0.60		Temp. /°C --->	30	Solvent --->	Ethanol
1140	0.22	0.39	0.40					
2280	0.33	0.29	0.30					
3600	0.38	0.23	0.22					
5820	0.46	0.17	0.18					
12420	0.52	0.09	0.11					
16620	0.54	0.08	0.09					
0	0	0.60	0.45		Temp. /°C --->	30	Solvent --->	Ethanol

<--- when prompted by yellow cells, add temperature and solvent

<--- initial conditions require entering 0 in time and [P] columns

Figure S2. A screenshot of the Data entry worksheet with representative data.

Conversions: reaction examples

Figure S3a. Up to five data sets can be shown on the conversion graph. An experimental summary is provided in the lower table from which to select data from. Experiments are automatically numbered in the order in which they were entered.

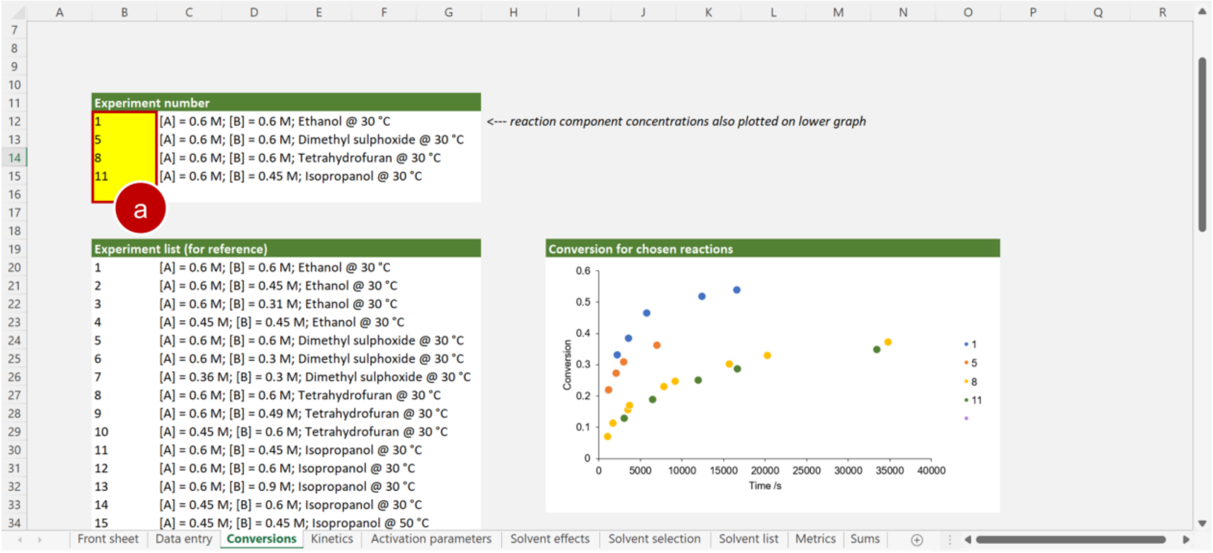

Figure S3. A screenshot of the Conversions worksheet.

Kinetics: blank sheet

Figure S4a. To determine the partial orders of the reaction, choose a data set. Each set of experiments (defined by the solvent and temperature) requires the partial orders of reaction to be determined before further analysis.
Figure S4b. It may be found that the order of reaction is independent of temperature, or independent of the solvent. This information can optionally be added and will shorten subsequent data entry (Microsoft 365 version only). If these options are changed after orders of reaction have been entered (see later), you will have to update this information.

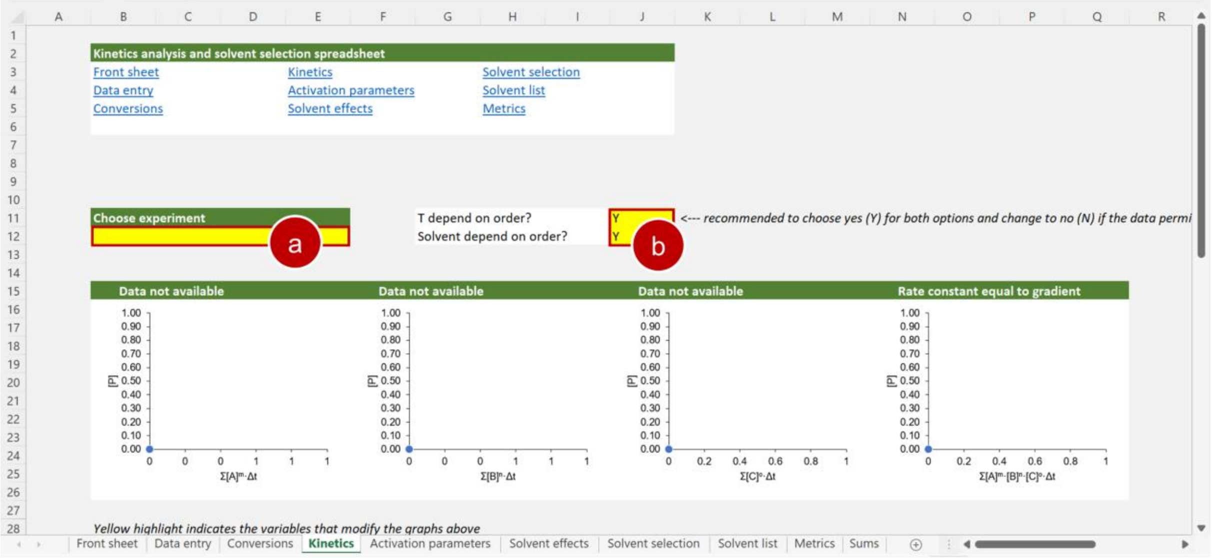

Figure S4. A screenshot of the Kinetics worksheet without data selected.

Please familiarise yourself with Variable Time Normalisation Analysis before continuing [5].

Kinetics: order determination

Figure S5a. In this example, experiments in dimethyl sulphoxide at 30 °C are analysed. In this example, the order of reaction is independent of temperature. The corresponding experiment is highlighted in the lower left table (simplified to ‘Dimethyl sulphoxide’ because the temperature is irrelevant, Microsoft 365 version only). If the incorrect orders of reaction are selected the graphs will not overlap and the right-side graph calculating the rate constant is not linear. Data is only shown in the charts if experiments have been conducted that vary the initial concentration of reactants.
Figure S5b. The rate constant is based on the order of reaction given. If an incorrect order of reaction is provided, then the rate constant will be incorrect.

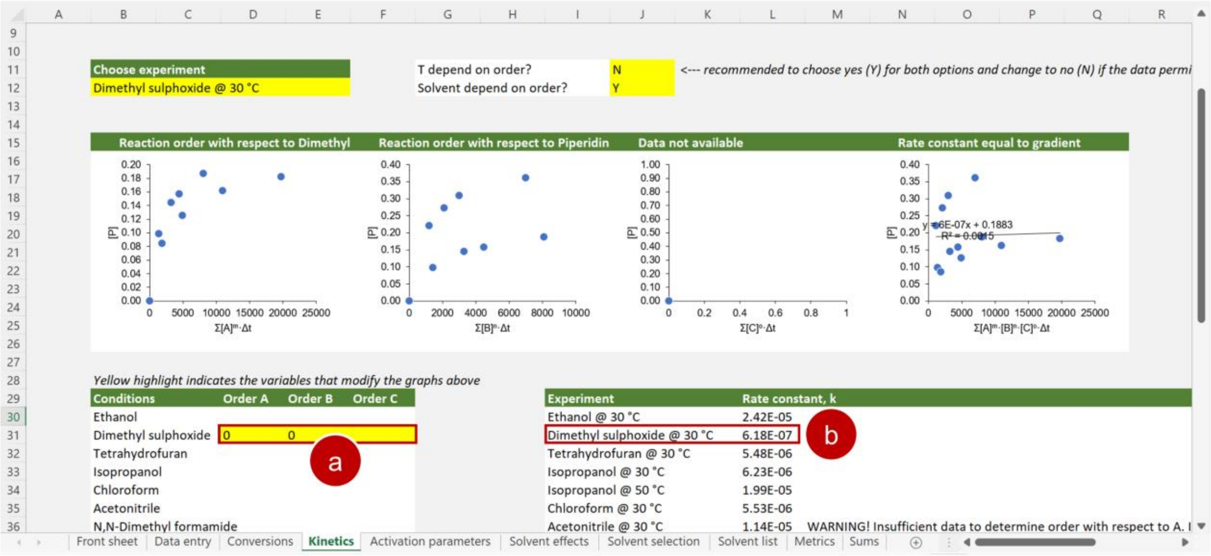

Figure S5. A screenshot of the Kinetics worksheet with incomplete data.

Figure S6a. The correct order of reaction with respect to A and B is added, and the graphs overlap. The partial order charts are not necessarily linear.
Figure S6b. The rate constant has updated, and the right-side graph shows a linear relationship, the gradient being the rate constant. If the correlation is poor, the chosen order of reaction is incorrect, or the raw data could be inaccurate.

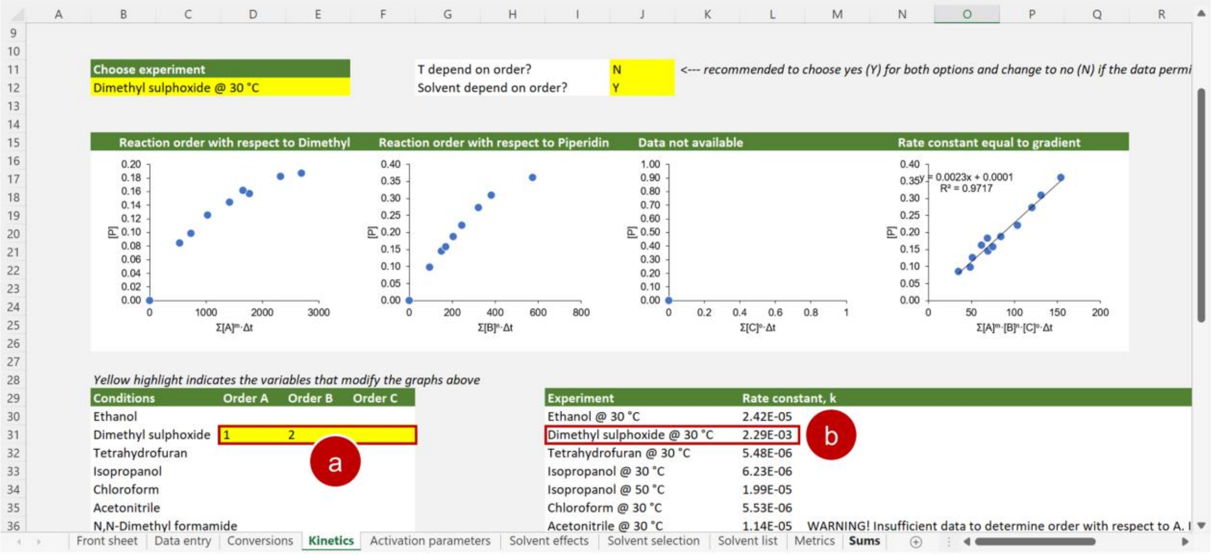

Figure S6. A screenshot of the Kinetics worksheet with the correct order of reaction.

Activation parameters: plotting the Eyring equation

Figure S7a. Select a solvent (only solvents with the appropriate data will be selectable).
Figure S7b. Data from relevant experiments will be displayed in this table. In this instance, four groups of experiments in ethanol are returned. To obtain accurate data please ensure the correct order of reaction was entered in the Kinetics worksheet.
Figure S7c. The enthalpy of activation and entropy of activation are given, as derived from the chart.

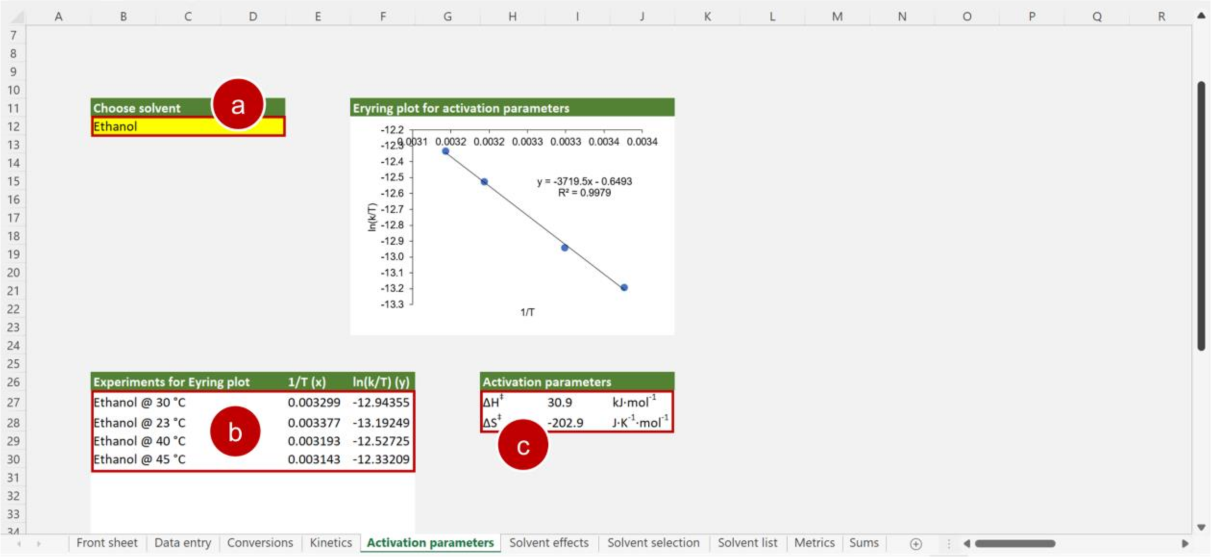

Figure S7. A screenshot of the Activation parameters worksheet with temperature variable kinetic data.

Solvent effects: blank sheet

Figure S8a. Rate constants can be correlated to the polarity of the solvent. To do so, select orders of reaction from those previously determined. It is assumed this will represent a single mechanism and exclude data from reactions with a different order of reaction. The final cell contains the desired temperature at which the model the solvent effect. This must be an experimental temperature chosen from the list that will match the selected order of reaction. At least 2 solvents are required that support the same order of reaction at the same temperature. If this is not true, no temperature options will be selectable.
Figure S8b. The coefficients of the solvent parameters are entered here to satisfy the linear solvation energy relationship described in Eq. S1.

Eq. S1 $\ln(k) = \mathrm{C} + \mathrm{A}\alpha + \mathrm{B}\beta + \mathrm{S}\pi^{*} + \mathrm{D}V_m$

In Eq. S1, C is a constant (automatically calculated), A is the coefficient of α (hydrogen bond donating ability), B is the coefficient of β (hydrogen bond accepting ability), S is the coefficient of π* (solvent polarity/polarisability), D is the coefficient of molar volume. In the reaction optimisier spreadsheet, molar volume is scaled by a factor of 100 to make the parameters of comparible magnitude.
You are able to enter relative quantities for the coefficients, which will be automatically corrected in the adjacent cells to estimate the solvent effect. This provides an estimate suitable for some users, but it is generally recommended to use the data analysis tools described subsequently.
Figure S8c. To accurately determine the solvent effect via a linear solvation energy relationship, the coefficients of the solvent parameters can be calculated using the Excel Solver function, or with a linear regression. The Kamlet-Taft parameters (α, β, π*), molar volumes, and experimental rate constants are found lower on this worksheet. This space is reserved for performing the linear regression. The Analysis ToolPak add-in is required.

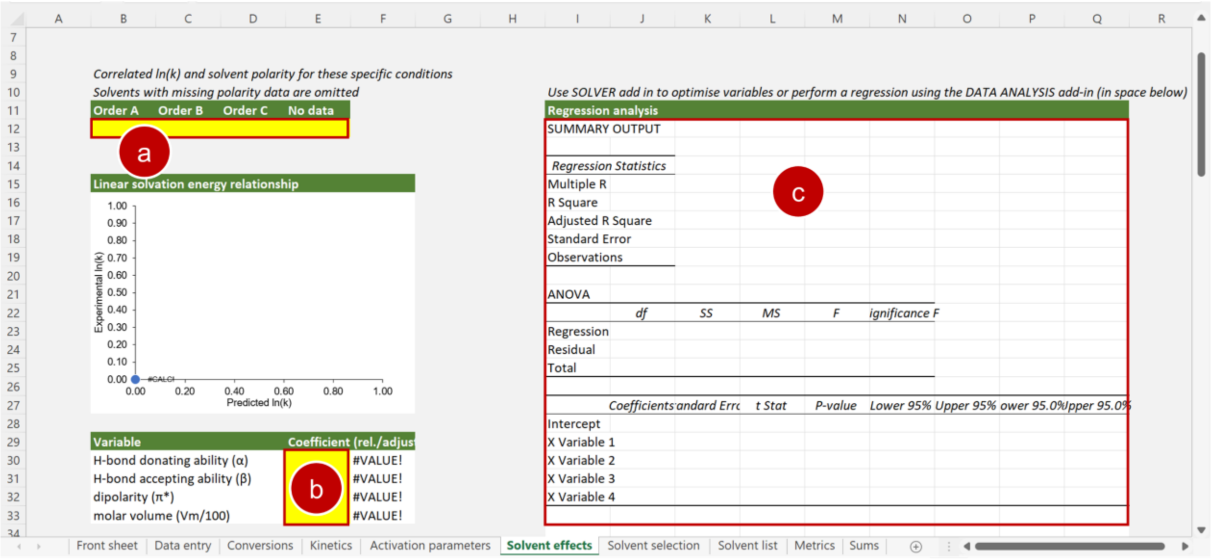

Figure S8. A screenshot of the Solvent effects worksheet with no data selected.

Solvent effects: linear solvation energy relationship

Figure S9a. The data set is chosen. For this example it is a trimolecular reaction at 30 °C.
Figure S9b. Solvent parameter coefficients have been chosen for the linear solvation energy relationship (Eq. S2).

Eq. S2 $\ln(k) = -11.89 + 0.21\alpha + 3.42\beta + 3.71\pi^* - \frac{0.04}{100}V_m$

A graph comparing the predicted ln(k) to the experimental ln(k) is now shown. The constant (of -11.89 in this instance) is given in cell C36.
Figure S9c. The linear regression output is shown. High P-values indicate the coefficient is not statistically significant. In this example, hydrogen bond donating ability (α) and molar volume (V_m) are not significant ($p > 0.9$), and the linear solvation energy relationship can be recalculated to exclude them if desired.

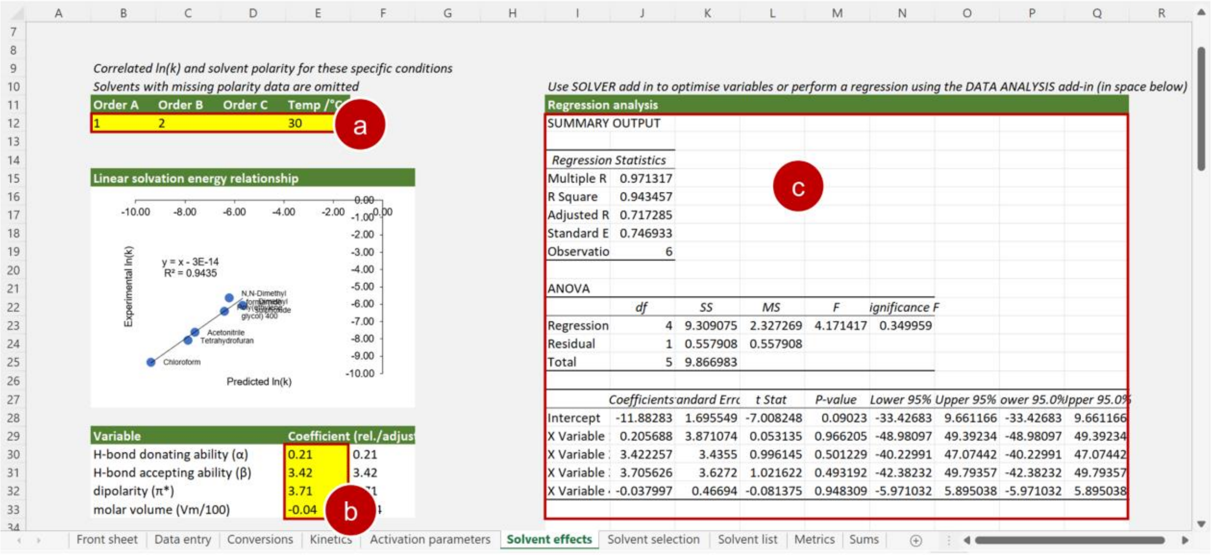

Figure S9. A screenshot of the Solvent effects worksheet with data for a linear solvation energy relationship.

Solvent selection: eligibility criteria

Figure S10a. Solvents will be ranked according to the predicted rate constant (following the linear solvation energy relationship, Eq. S2). Some reactive solvents may be excluded by the user. These will appear in grey, or it is possible using this menu to hide unsuitable solvents from the list below completely.
Figure S10b. The solvents with the highest predicted rate constants are shown. The greenness of those solvents is also provided, and so a solvent can be chosen based on performance and hazards. The greenness of the solvents is based on the CHEM21 solvent selection guide [6], where S represents safety, H represents health, and E represents environment. High scores (to a maximum of 10) are undesirable.

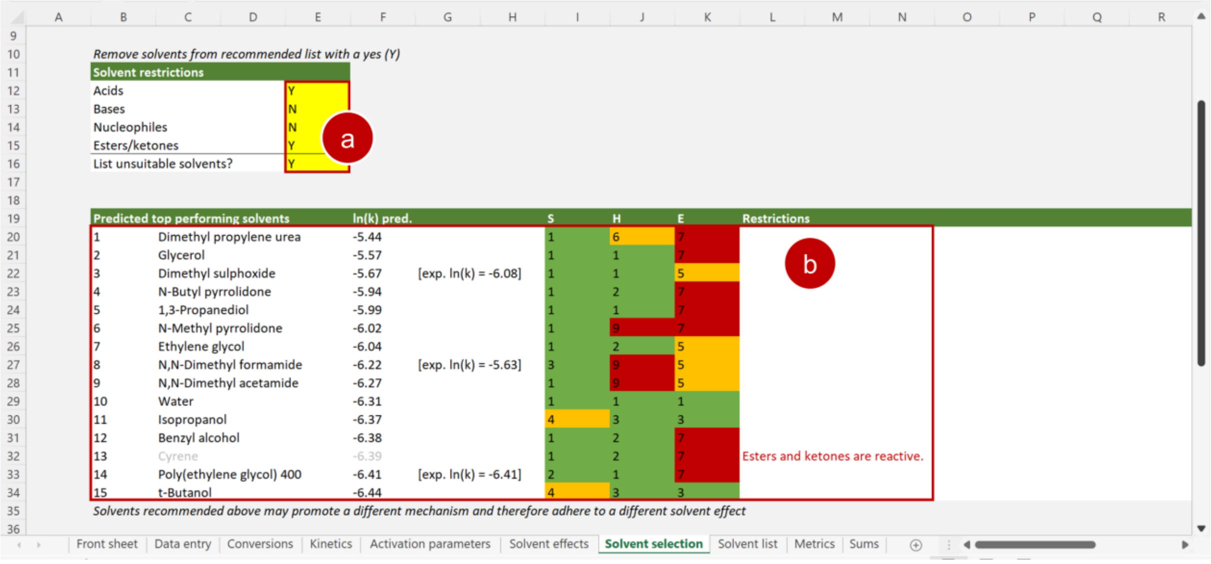

Figure S10. A screenshot of the Solvent selection worksheet with recommended solvents.

Metrics: blank sheet

Figure S11a. To evaluate a reaction with green metrics, add a solvent and temperature, and the order of reaction. This will allow conversions at specific times to be calculated. The functionality of this worksheet has limitations based on the availability of experimental data. These are noted by the annotations that update on the worksheet. If a linear solvation energy relationship has been inputted at the same temperature and reaction order as selected on this worksheet, any solvent can be selected (assuming solvent parameter data is available).
Figure S11b. If activation parameters have been obtained, these can be added and more flexibility with respect to the temperature selection is possible.
Figure S11c. The conversion at the selected time can be calculated. Experimental data will also be plotted on the accompanying chart if the solvent, temperature, and initial reagent concentrations match.
Figure S11d. The conversion obtained at a set point in the reaction is used to calculate yield and other metrics. The relative molecular mass and stoichiometry of the reaction must be added, as well as the volume of solvent used.

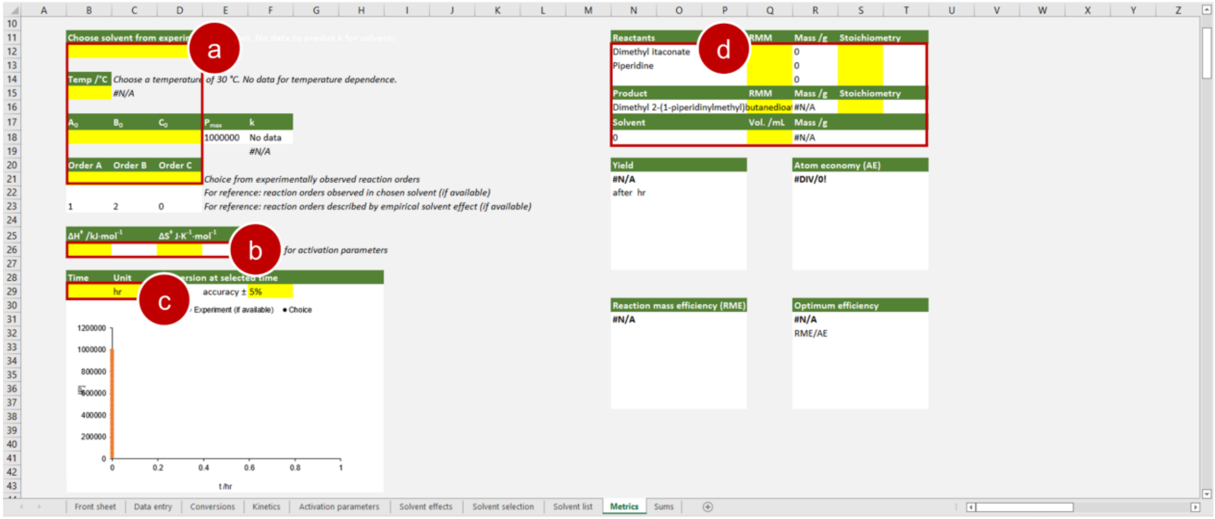

Figure S11. A screenshot of the Metrics worksheet, unfilled.

Metrics: Completed analysis

Figure S12a. Tetrahydrofuran at 30 °C with 0.6 M of both reactants has been selected. The chosen reaction order matches experiment and the conditions of the linear solvation energy relationship.
Figure S12b. At 12 hours, the predicted conversion is 68%. Experimental data is also plotted (unfilled blue circles). In reactions with significant side products, the calculated conversion will be inaccurate.
Figure S12c. Green metrics have been calculated [7]. The atom economy is 100% because this is an addition reaction. Reaction mass efficiency (RME) and optimum efficiency are also provided.

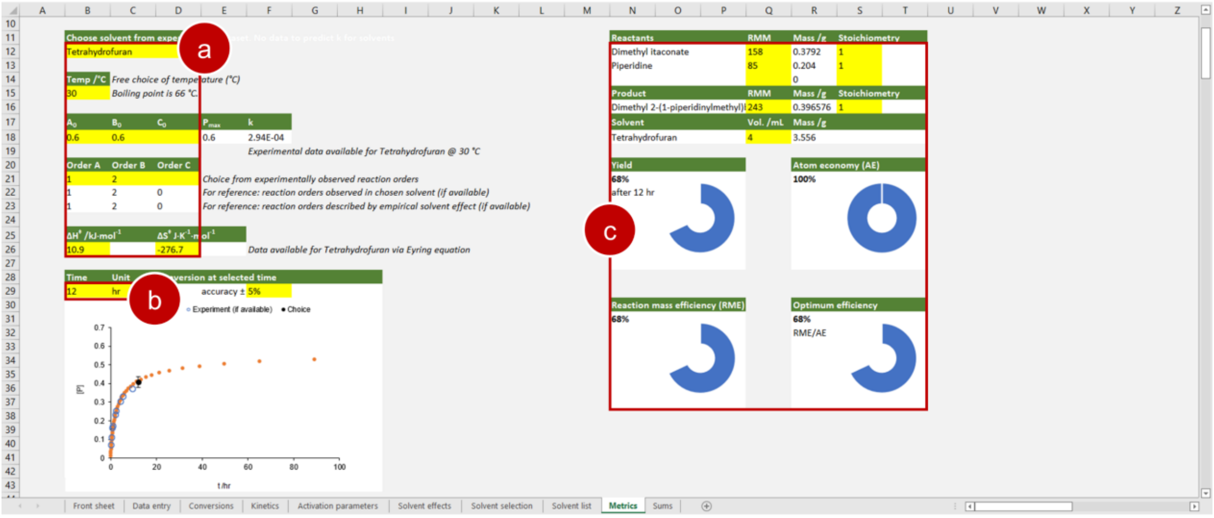

Figure S12. A screenshot of the Metrics worksheet with data corresponding to an experiment.

2. Experimental methods

2.1 Aza-Michael reaction

The data set used to train the spreadsheet was previously published [1]. New experiments were performed according to the same method. Dimethyl itaconate (0.633 g, 4 mmol) and benzyl benzoate (0.0200 g) in ethanol or dimethyl sulphoxide (2 mL) was preheated to 30 °C. Piperidine (0.341 g, 4 mmol) was added and the reaction mixture stirred for 2 hours. The conversion to dimethyl 2-(1-piperidinylmethyl)butanedioate was analysed by ^{1}H NMR spectroscopy (400 MHz, in $CDCl_3$). The concentration of compounds was calculated from the known concentration of internal standard benzyl benzoate (CH_2 signal).

2.2 Michael reaction

The data set used to train the spreadsheet was obtained by previously published methods [4]. Dimethyl malonate (0.158 g, 1.2 mmol) and trans-chalcone (0.208 g, 1.0 mmol) were dissolved in the chosen solvent (5 mL), potassium phosphate (0.036 g, 0.17 mmol) was added, and the resulting suspension stirred at room temperature for up to 24 hours. Aliquots were dissolved in acetone, filtered, and analysed by a Hewlett-Packard 6890 series gas chromatograph (GC) using an FID (Flame Ionisation Detector) and a ZB-HT5 fused silica column (30 m x 0.25 mm x 0.25 μm). The inlet was set at a temperature of 300 °C with a split ratio of 60, and an initial oven temperature of 50 °C before ramping at 30 °C min^{-1} to 300 °C, which was then held for 5 minutes.

2.3 Amidation

The data set used to train the spreadsheet was obtained by previously published methods [3]. New experiments were performed in the same way. To a solution of 4-phenylbutanoic acid (1.806 g, 11 mmol) in toluene (10 mL) preheated to 384 K was added benzylamine (0.589 g, 5.5 mmol). Conversion to *N*-benzyl-4-phenylbutanamide was analysed using ^{1}H-NMR spectroscopy (400 MHz, in $CDCl_3$) of reaction aliquots at hourly intervals.

2.4 Data interpretation

Kinetic analysis was performed using Variable Time Normalisation Analysis in the manner described in the literature [5]. Solvent effects were determined using linear solvation energy relationships. Correlations were found using the regression function of Microsoft Excel. Variables were generally excluded if p-values were above 0.1.

References

1 Day, D.M.; Farmer, T.J.; Sherwood, J.; Clark, J.H. An experimental investigation into the kinetics and mechanism of the aza-Michael additions of dimethyl itaconate. *Tetrahedron* **2022**, *121*, 132921. https://doi.org/10.1016/j.tet.2022.132921

2 Clark, J.H.; Fitzpatrick, E.M.; Macquarrie, D.J.; Pfaltzgraff, L.A.; Sherwood, J. p-Cymenesulphonic acid: an organic acid synthesised from citrus waste. *Catal. Today* **2012**, *190*, 144–149. https://doi.org/10.1016/j.cattod.2011.12.007

3 Clark, J.H.; Macquarrie, D.J.; Sherwood, J. A quantitative comparison between conventional and bio-derived solvents from citrus waste in esterification and amidation kinetic studies. *Green Chem.* **2012**, *14*, 90–93. https://doi.org/10.1039/C1GC16299C

4 Sherwood, J.; Granelli, J.; McElroy, C.R.; Clark, J.H. A method of calculating the Kamlet–Abboud–Taft solvatochromic parameters Using COSMO-RS. *Molecules* **2019**, *24*, 2209. https://doi.org/10.3390/molecules24122209

5 Burés, J. Variable time normalization analysis: general graphical elucidation of reaction orders from concentration profiles. *Angew. Chem. Ind. Ed.* **2016**, *55*, 16084–16087. https://doi.org/10.1002/anie.201609757

6 Prat, D.; Wells, A.; Hayler, J.; Sneddon, H.; McElroy, C.R.; Abou-Shehada, S.; Dunn, P.J. CHEM21 selection guide of classical- and less classical-solvents. *Green Chem.* **2016**, *18*, 288–296. https://doi.org/10.1039/C5GC01008J

7 McElroy, C.R.; Constantinou, A.; Jones, L.C.; Summerton, L.; Clark, J.H. Towards a holistic approach to metrics for the 21st century pharmaceutical industry. *Green Chem.* **2015**, *17*, 3111–3121. https://doi.org/10.1039/C5GC00340G
